# Evaluation and Surgical Management of Adult Degenerative Scoliosis Associated With Lumbar Stenosis

**DOI:** 10.1097/MD.0000000000003394

**Published:** 2016-04-18

**Authors:** Guodong Wang, Xingang Cui, Zhensong Jiang, Tao Li, Xiaoyang Liu, Jianmin Sun

**Affiliations:** From the Department of Spine, Shandong Provincial Hospital Affiliated to Shandong University, Jinan, Shandong, China.

## Abstract

Adult degenerative scoliosis associated with lumbar stenosis has become a common issue in the elderly population. But its surgical management is on debating. The main issue condenses on the management priority of scoliosis or stenosis.

This study is to investigate surgical management strategy and outcome of adult degenerative scoliosis associated with lumbar stenosis.

Between January 2003 and December 2010, 108 patients were admitted to the authors’ institution for adult degenerative scoliosis associated with lumbar stenosis. They were divided into 3 groups based on the symptom. Then the surgical management was carried out. The clinical outcome was evaluated according to the Oswestry Disability Index (ODI) and Scoliosis Research Society-22 score (SRS-22 score) at follow up. Group 1 was with primary lumbar stenosis symptom, local decompression and short fusion were performed. Group 2 was with compensated spinal imbalance symptom, local decompression of the symptomatic spinal stenosis and short fusion were performed. Group 3 was with primary spinal imbalance, correction surgery and long fusion were performed.

For Group 1, the ODI scores declined from 62.5 ± 4.2 preoperatively to 21.8 ± 2.5 at final follow up, the SRS-22 scores decreased from 44.8 ± 3.2 preoperatively to 70.9 ± 6.0 at final follow up. For Group 2, the ODI and SRS-22 scores were 73.4 ± 8.4 and 40.8 ± 8.5 before the surgery, declined to 22.4 ± 4.2 and 73.2 ± 7.9 at final follow up. For Group 3, the ODI and SRS-22 scores were 73.4 ± 4.9 and 45.3 ± 6.4 before surgery, declined to 30.4 ± 8.9 and 68.8 ± 8.1 at final follow up.

It was effective to perform decompression and short fusion for Group 1 and correction surgery and long fusion for Group 3. For Group 2, the compensated imbalance symptom was always provoked by the symptomatic lumbar stenosis. The cases in the Group 2 got well clinical improvements after local surgical intervene on the symptomatic spinal stenosis and short fusion, leaving the deformity untreated.

## INTRODUCTION

Adult degenerative scoliosis (ADS), also named as de novo scoliosis,^1^ represents a structural curve developed after skeletal maturity without previous scoliosis history.^2,3^ It differs from other kinds of structural scoliosis and nonstructural scoliosis.^4^ ADS is caused by asymmetric degeneration of spinal motion segments, thus often accompanies with lumbar stenosis, rotational olisthesis, and lumbar kyphosis.^1^

Nowadays, ADS associated with lumbar stenosis has become a common issue in elderly population.^3,5^ There are 2 common symptoms in ADS cases^6^: the lumbar stenosis symptoms and the spinal imbalance symptoms. The former one includes neurological claudication and radiculopathy. The latter one is the mechanical axial pain in nature and the incapacity to stand upright, mainly caused by an aggressive deformity and weak back muscle.

Sometimes the imbalance symptoms are provoked by the lumbar stenosis.^4^ It is considered as the compensated imbalance, which is different from the primary imbalance. The compensated imbalance occurs consequently to the symptomatic lumbar stenosis and aggravated within a few days. It mainly presents as the incapacity to stand upright. The radical pain caused by the lumbar stenosis could mask the mechanical axial pain.

Thus, the symptom spectrum of ADS associated with lumbar stenosis consists of the primary stenosis symptom, the compensated imbalance symptom, and the primary imbalance symptom.

Based on the symptom spectrum, the ADS cases were divided into 3 groups and different surgical managements were applied. In this study, the outcome and effectiveness of the surgical treatments of the ADS patients were investigated.

## METHODS

### Subjects

Permission to conduct this retrospective study was obtained from the hospital ethics committee.

Between January 2003 and December 2010, 108 consecutive patients with ADS associated with lumbar stenosis were included in this study. They all underwent surgical treatment by the same surgeon in a university affiliated hospital.

Inclusion criteria:^7^ ADS with lumbar stenosis, with at least 2 years follow up.^8^ The curve of the scoliosis was >30° according to the Cobb method, or >15° with lumbar (thoracolumbar) kyphosis, or with documental progression more than 10° per y (either coronal or sagittal plane).

Exclusion criteria^4^: Nonstructural scoliosis, in which the curve had no rotation component, including postural, hysterical, sciatic, inflammatory, and compensatory scoliosis. The other kinds of structural scoliosis, including congenital, idiopathic neuromuscular, traumatic and iatrogenic scoliosis. Patients with follow up <2 years.

### Group Category and Surgical Strategy

**FIGURE 1:**
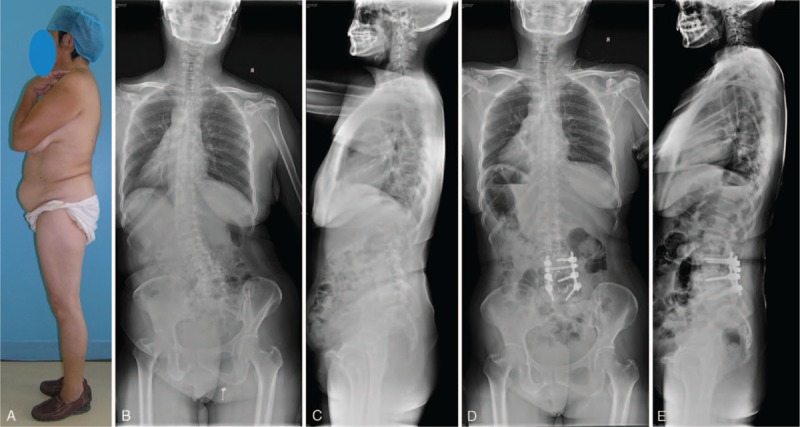
(A) The patient could stand erectly without any support. She belonged to Group 1. Figures (B/C) showed a well sagittal balance. (D/E) The patient underwent a 2-level decompression and fusion. Transforaminal lumbar intervertebral fusion was performed. Good sagittal balance was maintained.

Group 1: cases with primary stenosis symptom. Decompression of the symptomatic spinal stenosis and short fusion was performed (Figure 1).

**FIGURE 2:**
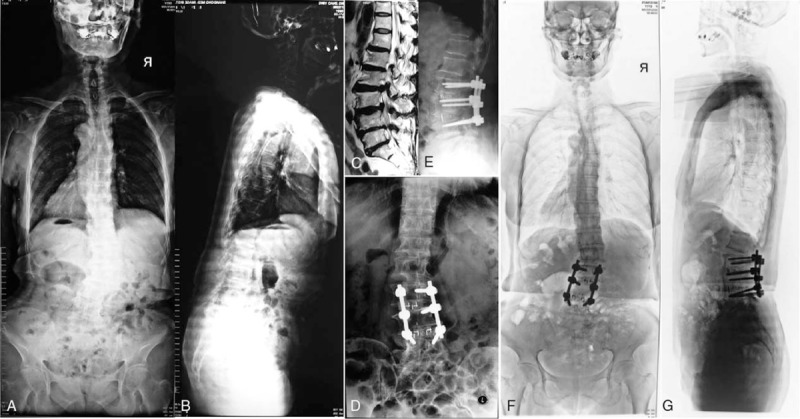
(A/B) A 65-year-old female patient belonged to Group 2, with a lumbar curve as 30°, lumbar lordosis as 15°, thoracic lordosis as 5°. (C) Magnetic resonance imaging showed disc hernia occurred at L2/3 and L3/4. (D/E) The patient underwent decompression and 2 levels fusion (TLIF). (F/G) At 3-year follow-up, the balance was well maintained. TLIF = transforaminal lumbar intervertebral fusion.

Group 2: cases with compensated imbalance symptom. Local decompression of the symptomatic spinal stenosis was performed. Short fusion after decompression was performed too (Figure 2).

**FIGURE 3:**
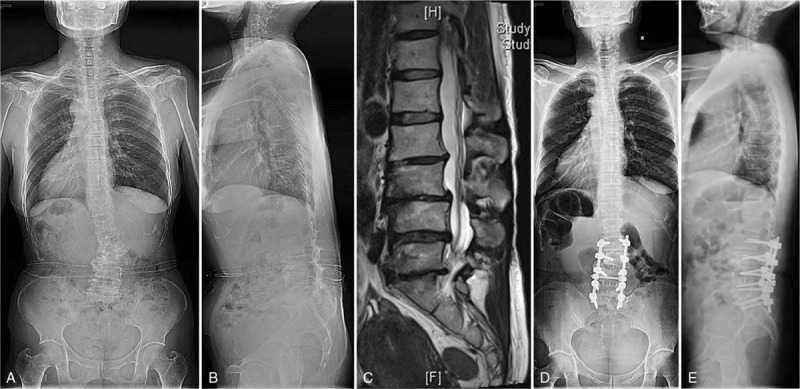
(A/B) A 55-year-old female patient belonged to Group 3. The lumbar curve was 22°, the lumbar kyphosis was 20°. (C) Magnetic resonance imaging showed spinal stenosis occurred at L3/4, 4/5, L5/S1. (D/E) The patient underwent correction and long fusion from L2 to S1. The lumbar curve was 6°, lumbar lordosis was 18° at the final follow-up.

Group 3: cases with primary imbalance symptom, correction surgery, and long fusion were performed. Thoracic spine was included in fusion level when it was hyper-kyphosis. Pedicle subtraction osteotomy^9^ or Ponty osteotomy^10^ was performed when it is necessary to make enough lumbar lordosis (Figure 3).

### Evaluation Methods

Detailed history was obtained including the main symptoms and previous history. Detailed physical and neurological examinations were performed before the surgery and at follow up. The lumbar stenosis symptom includes neurological claudication and radiculopathy. The primary imbalance symptom is the incapacity to stand upright, accompanied with the mechanical axial back pain, aggravated by lifting things in front of body, relieved through supporting by arm in front. The axial pain aggravated gradually by time till falling collapse. The compensated imbalance symptom occurred consequently to the lumbar stenosis and aggravated within days. It mainly presented as the incapacity to stand upright. The mechanical axial pain was masked by the radical pain caused by the lumbar stenosis.

Scoliosis Research Society-22 (SRS-22)^11^and Oswestry Disability Index (ODI)^12^ were used to evaluate the pain and function before and after the surgery.

Radiological examinations were also obtained before surgery, including full spine anteroposterior (AP) and lateral radiographs, dynamic radiographs including lateral bending, extreme lumbar flexion and extension, 3-dimensional computer tomography (CT) scan, and magnetic resonance imaging (MRI) scan. If necessary, lumbar nerve root blocking was used to make sure which segment caused the radicular pain. After surgery and at follow up, full spine AP and lateral radiographs were carried out.

One author as the independent observer finished the evaluation procedure in a blinded manner. Statistical data were analyzed using Statistical Package for Social Sciences (SPSS, version 20) software. Comparison between the 3 groups was explored with ANOVA or Pearson Chi-squared test for the demographic data, the ODI scores, the SRS-22 scores, and the radiological parameters. The patients who died during the follow-up period were treated as censored. *P* value <0.05 was regarded as statistically significant and 2-sided tests were used during all the analyses.

## RESULTS

A total of 108 consecutive ADS patients were included between 2003 and 2010. There were 35 cases in Group 1, 34 cases in Group 2, and 39 cases in Group 3. All the cases underwent surgical treatment by the same surgeon in a university affiliated hospital. There were 34 male and 74 female. The mean age was 62 ± 8 years old (range: 47–78). All the surgeries were performed through posterior approach. The mean surgery time was 119 ± 66 min (range: 40–328). The mean blood loss was 977 ± 669 mL (range: 209–2900). The mean follow-up time was 34 months (range: 26–71) (Table 1).

**TABLE 1:**
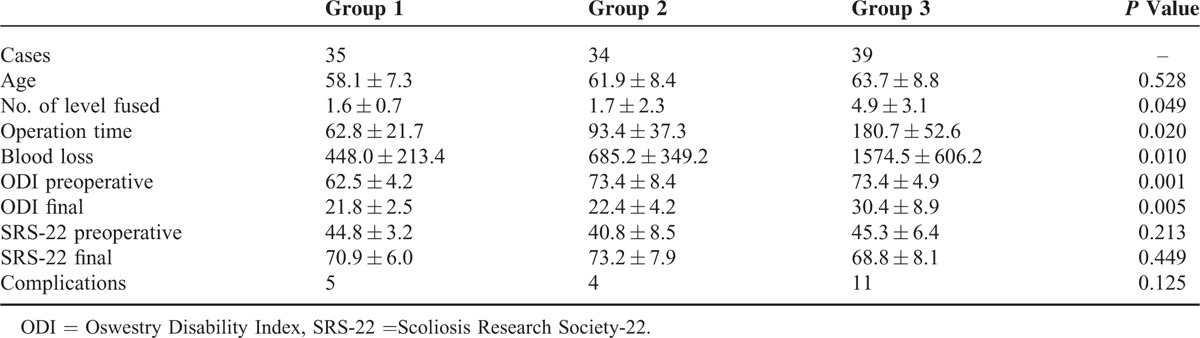
Clinical Parameters of Adult Degenerative Scoliosis Associated With Spinal Stenosis

In Group 1, the average preoperative (pre-op) lumbar scoliosis was 21.8 ± 5.8°, at final follow up 17.2 ± 4.7°; pre-op lumbar lordosis was 17.3 ± 13.2°, at final follow up 19.9 ± 13.3°; and pre-op thoracic kyphosis was 13.4 ± 14.7°, at final follow up 23.3 ± 10.1°. The average pre-op C7 plumb at the coronal plane was 2.8 ± 2.1 cm, at final follow up was 0.7 ± 0.5 cm; the pre-op C7 plumb at sagittal plane was 0.8 ± 0.7 cm, at final follow up was 1.2 ± 1.4 cm (Table 2). Pre-op ODI was 62.4 ± 4.2, at final follow up was 21.8 ± 2.5 and pre-op SRS-22 was 44.8 ± 3.2, at final follow up was 70.9 ± 6.0. Five patients had complications, including 1 with early infection, 3 with epidural hematoma, and 1 with pseudoarthrosis.

**TABLE 2:**
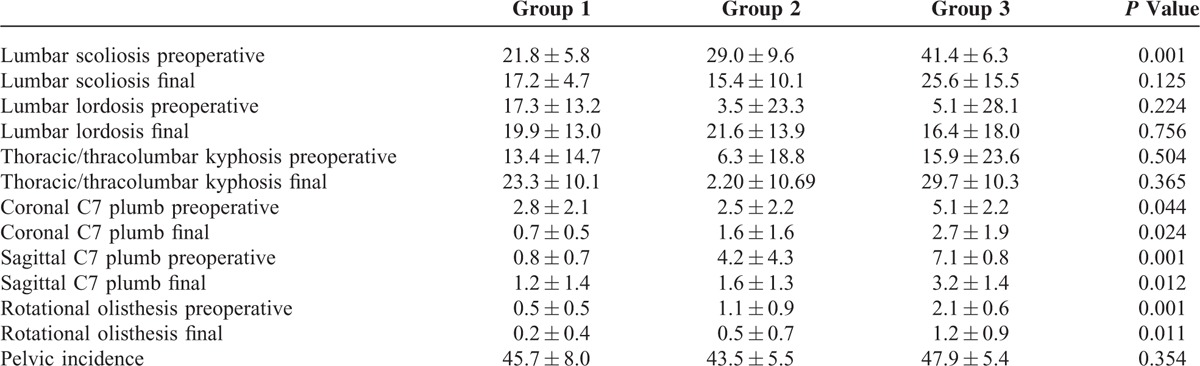
Radiological Parameters of Adult Degenerative Scoliosis Associated With Spinal Stenosis

In Group 2, pre-op lumbar scoliosis was 29.0 ± 9.6°, at final follow up 15.4 ± 10.1°; pre-op lumbar lordosis was 3.5 ± 23.3° (−40 to 34), at final follow up 21.6 ± 13.9° (9–42); and pre-op thoracic kyphosis was 6.3 ± 18.8° (−19 to 33), at final follow up 2.2 ± 10.7° (−7 to 41). The average pre-op C7 plumb at the coronal plane was 2.5 ± 2.2 cm, at final follow up was 1.6 ± 1.6 cm; the pre-op C7 plumb at sagittal plane was 4.2 ± 4.3 cm, at final follow up was 1.6 ± 1.3 cm (Table 2). Pre-op ODI was 73.4 ± 8.4, at final follow up was 22.4 ± 4.2 and pre-op SRS-22 was 40.8 ± 8.5, at final follow up was 73.2 ± 7.9. One patient experienced respiratory distress syndrome, 2 patients with pseudoarthrosis, 1 underwent early infection.

In Group 3, pre-op lumbar scoliosis was 41.4 ± 6.3°, at final follow up 25.6 ± 15.5°; pre-op lumbar lordosis was 5.1 ± 28.1° (−47 to 37), at final follow up was 16.4 ± 18.0 (−9 to 35); and pre-op thoracic/thoracolumbar 15.9 ± 23.6 (−23 to 36), at final follow up was 29.7 ± 10.3 (14–41). The average pre-op C7 plumb at the coronal plane was 5.1 ± 2.2 cm, at final follow up was 2.8 ± 1.9 cm and the pre-op C7 plumb at sagittal plane was 7.1 ± 0.8 cm, at final follow up was 3.2 ± 1.4 cm (Table 2). Pre-op ODI was 73.4 ± 4.9, at final follow up was 30.4 ± 8.9 and pre-op SRS-22 was 45.3 ± 6.4, at final follow up was 68.9 ± 8.1. Eleven patients underwent complications, included 2 with transient ischemic heart diseases, 1 with respiratory distress syndrome, 2 with transient neurological deficit, 2 with superficial wound infection, 3 with hypostatic pneumonia, and 2 with pseudoarthrosis.

## DISCUSSION

Patients with ADS are usually elderly patients accompanied with lumbar stenosis.^13^ The reason caused ADS is the asymmetric degeneration of spinal motion segments, including intervertebral disc, annulus fibrosus, ligmentsflavum, and the facet joint.^1^ The degeneration generates not only the scoliosis but also the lumbar stenosis.^14^ The ADS and lumbar stenosis cause symptoms, respectively and interactively.^4^

Chen's report^6^ divided the ADS symptoms into 2 main kinds: the spinal stenosis associated symptom and the spinal deformity symptom. The stenosis symptom is the neurological pain and the deformity symptom is the axial pain caused by mechanical factors. Then they deduced surgical strategy from this 2-group category method. Decompression is performed on cases with neurological pain caused by primary lumbar stenosis and correction surgery was operated on cases with mechanical pain caused by degenerative deformity.

Lenke and Silva make surgical strategy according to the patient's main symptom and medical condition.^4^ The most critical point is the pain's nature. The purely axial pain is mainly correlated with sagittal imbalance. The radical pain is generated by spinal stenosis. They also pointed out that the sagittal imbalance-related pain is not relieved by forward posture, unless the patient sits or stands with the trunk supported by the arms.

The lumbar stenosis symptom consists of the stenosis generated pain and the neurological claudication. The stenosis generated pain is mainly caused by the nerve compression, due to the central or lateral spinal stenosis.^1^ Ploumis et al^15^ pointed out that it was important to distinguish the neurological claudication from the vascular claudication. The pain of neurological claudication can be relieved by forward posture (like bicycling), but the vascular claudication is relieved by standing still.

The imbalance symptom consists of the axial pain and the incapacity to stand upright. The sagittal imbalance-related pain appears as the muscle pain because of the fatigue of spinal muscles and it is attributed to the loss of lumbar lordosis. The pain at concave side might be cause by joint arthritis and degenerative changes of disc.^16^ Glassman et al^17^ supported this point by reviewing 752 adult scoliosis patients.

However, not all the imbalance symptom is primary.^6^ The compensated imbalance occurs consequently to the symptomatic stenosis at the patient without primary imbalance. The might-be reason is that the spinal muscle is restricted due to the painful spinal stenosis,^18^ because the extension would deteriorate the lumbar stenosis. The patient cannot stand upright because no spinal muscle acts, unless he or she supports his or her trunk by arms. Then the compensated imbalance occurs. It mainly presents as the incapacity to stand upright. There are 2 kinds of pain for the patients with the compensated imbalance, the radical pain and the axial pain. The extent of the 2 kinds of pain is different. The radical pain caused by the lumbar stenosis is the major issue, often masks the axial pain.

Thus the etiology of symptoms in ADS patients are lumbar stenosis, primary imbalance, and compensated imbalance.^19^ The stenosis generated pain is resolved by decompression and short fusion. The primary imbalance-related pain is treated by correction surgery and long fusion. There are debating on the treatment of the compensated imbalance. Some reports point out that the decompression and short fusion is the better choice, because the medical condition of the elderly patients should be taken account in.

The medical condition of the elderly patients with ADS is an important issue. For the elderly patients with ADS associated with lumbar stenosis, the ideal goal is keeping the surgical intervene as minimal as possible. Albeit several authors reported that correction surgery with long fusion generates better outcome and satisfaction,^1,20^ the accompanying medical comorbidities on the elderly population get more attention nowadays. Ploumis et al^15^ reported that it should not be overlooked. Lenke and Silva divided the surgeries of ADS into 6 levels.^4^ They advocated to the lowest level surgery to solve the patients’ symptom.

In this study, the symptom spectrum is divided into 3 groups and then all the ADS cases were divided accordingly. The surgical strategy is deduced according to the 3-group category.

Patients in Group 1 only complain the lumbar stenosis symptom. Patients in Group 2 complain symptomatic spinal stenosis and the compensated imbalance. Local decompression and short fusion relieve the pain of patients in Groups 1 and 2. In order to perform the local decompression and short fusion surgery, the priority job is to find out the responsible segment, since almost all the lumbar segments are not good on radiographs of the elderly patients. It is helpful to identify the responsible segment through checking the specific area where the radicular pain distributed and thin-layer CT or MRI scanning of the lumbar intervertebral foramina. Lumbar nerve root block is a common technique to make sure the responsible segment. If the responsible segment is instable, especially with rotational olisthesis, simple decompression might cause aggravation of rotational olisthesis and iatrogenic instability. Transforaminal lumbar intervertebral fusion is the fantasy perfection. For the responsible segments outside the apical area, simple decompression might be enough.

Patients in Group 3 complain primary imbalance symptom. Correction surgery and long fusion are usually needed. The decreased thoracic kyphosis is an indicator of strengthened spinal muscle, fusion stopping at T12 or L1 might be good. Otherwise, a long fusion to include the thoracic kyphosis region is necessary to prevent proximal junctional kyphosis. Osteotomy is helpful to gain enough lumbar lordosis. Smith–Peterson Osteotomy, Ponte osteotomy, or pedicle subtraction osteotomy are available. Vertebrae column resection is not recommended because of more blood loss and longer surgery time.^4^ According to Aebi's report,^21^ patients older than 65 years and those with major morbidities are supposed risky to perform correction surgery. For those ADS patients who could not bear correction surgery, it is very critical to communicate with patient to make sure whether he or she is satisfied with the surgery only intervene the symptomatic spinal stenosis but leaving imbalanced symptom untreated. Brace is a method to alleviate the imbalance symptom, but it cannot stop the progression of ADS and plays a bad role against functional exercise of back muscle.^22^

## CONCLUSION

ADS associated with lumbar stenosis can be divided into 3 groups based on the symptom. Group 1 is with primary lumbar stenosis symptom, Group 2 with compensated spinal imbalance symptom, and Group 3 with primary spinal imbalance. It is effective to perform decompression and short fusion for Group 1 and correction surgery and long fusion for Group 3. For Group 2, the compensated imbalance symptom is always provoked by the symptomatic lumbar stenosis. The cases in the Group 2 get well clinical improvements after local surgical intervene on the symptomatic spinal stenosis and short fusion, leaving the deformity untreated.
